# Supplementary material for: β2-subunit alternative splicing stabilizes Cav2.3 Ca2+ channel activity during continuous midbrain dopamine neuron-like activity
Source: eLife. 2022 Jul 6;11:e67464. doi: 10.7554/eLife.67464 (PMC9307272; doi:10.7554/eLife.67464)
Supplement: Supplementary file 1. — For details see methods. [file elife-67464-supp1.docx]

**Supplemetary File 1**

| **gene symbol** | **name** | **cellular function** | **assay ID** | |  |
| --- | --- | --- | --- | --- | --- |
| *Actb* | beta-actin | cytoskeletal protein | | Mm00607939_s1 | |
| *B2m* | beta-2-microglobulin | immunity protein | | Mm00437762_m1 | |
| *Gapdh* | glyceraldehyde-3-phosphate dehydrogenase | oxidoreductase | | Mm99999915_g1 | |
| *Hprt1* | hypoxanthine phosphoribosyl-transferase 1 | transferase | | Mm00446968_m1 | |
| *Tbp* | tata box binding protein | transcription factor | | Mm00446973_m1 | |
| *Tfrc* | transferrin receptor | receptor | | Mm00441941_m1 | |
| *Sdha* | succinate dehydrogenase complex, subunit A | oxidoreductase | | Mm01352363_m1 | |
