# Supplementary material for: β2-subunit alternative splicing stabilizes Cav2.3 Ca2+ channel activity during continuous midbrain dopamine neuron-like activity
Source: eLife. 2022 Jul 6;11:e67464. doi: 10.7554/eLife.67464 (PMC9307272; doi:10.7554/eLife.67464)
Supplement: Supplementary file 2. — Additional SalI and HindIII restriction enzyme sites (see Methods) are underlined; the reverse (rev) primer used for standard template cloning including the HindIII restriction site was the same for β2a-e subunit variants; rev, reverse; fwd, forward. [file elife-67464-supp2.docx]

**Supplemetary File 2**

| **β-subunit** | **genbank**  **accession**  **no.** | **assay ID or sequences**  **for custom assay** | **exon**  **boundary** | **primer sequences for standard template cloning** | **fragment**  **length (bp)** |
| --- | --- | --- | --- | --- | --- |
| β1 | NM_031173.4 | Mm00518940_m1 | 2/3 | Fwd: 5´-ATATGTCGACTCTCCATGGTCCAGAAGAGC-3´  Rev: 5´-CTTGAAGCTTGTACTTCTCCTTGATGTGTA-3´ | 455 |
| β2 | NM_023116.4 | Mm01333550_m1 | 13/14 | Fwd: 5´-ATATGTCGACAATGTCAGATACAGCGCAGC-3  Rev: 5'-CTTGAAGCTTTGTTACACTGTTTGCACTGG-3’ | 419 |
| β3 | NM_007581.3 | Mm00432233_m1 | 1/2 | Fwd: 5´-ATATGTCGACCCCATGTATGACGACTCCTACG-3  Rev: 5'-CTTGAAGCTTACAGTAGCTGACATTGGTCCTCAC-3’ | 236 |
| β4 | NM_146123.3 | Mm00521623_m1 | 11/12 | Fwd: 5´-ATATGTCGACGCTGATTAAGTCCAGAGGAAAGTC-3  Rev: 5'-CTTGAAGCTTTGTCTCATTCGCTGACTCTGTAAT-3’ | 308 |
| β2a | XM_011238946.2 | Fwd: 5´-CACGGTGCCGCTTGGT-3´  Rev: 5´-TGCATGAAGAGGTGGCAGAA-3´  Probe: 5´-AAGCCACGCTCTGAC-3´ | 5´-UTR-ex2B | Fwd: 5´-ATATGTCGACTGGACAGTGGCCGTGACGAG-3  Rev: 5´-CTTGAAGCTTCACAGCCTTCTTTAACCAGC-3´ | 440 |
| β2b | NM_001252533.1 | Fwd: 5´-TTACACATCTCAAACTTCAGGGAAAA-3´  Rev: 5´-CCAGCTAAAGGTGGCTTTGC-3´  Probe: 5´-CGGAGCCCGTGCGA-3´ | 5´-UTR | Fwd: 5´-ATATGTCGACCGTTAGAAAGTCATGAAGTC-3´  Rev: 5´-CTTGAAGCTTCACAGCCTTCTTTAACCAGC-3´ | 523 |
| β2c+d | XM_006497314.3  NM_023116.4 | Fwd: 5´-AAAGGCTCGGATGGAAGCA-3´  Rev: 5´-CCCTGGCGGACAAAACTGT-3´  Probe: 5´-ATCGTCAGACACTACCTC-3´ | ex2A-3 | Fwd: 5´-ATATGTCGACTTGCCGATGGTCCAAAGCGAC-3´  Rev: 5´-CTTGAAGCTTCACAGCCTTCTTTAACCAGC-3´ | 519 |
| β2e | NM_001309519.1 | Fwd: 5´-GGGAGGAAGGCTGAAGAGTTC-3´  Rev: 5´-GGGCGGCTGGTGTAGGA-3´  Probe: 5´-ACATCTGTGGTTCGGC-3´ | ex2D-3 | Fwd: 5´-ATATGTCGACCTACCCGGCTCATGAAGGCCACCTGG-3´  Rev: 5´-CTTGAAGCTTCACAGCCTTCTTTAACCAGC-3´ | 392 |
