# Supplementary material for: β2-subunit alternative splicing stabilizes Cav2.3 Ca2+ channel activity during continuous midbrain dopamine neuron-like activity
Source: eLife. 2022 Jul 6;11:e67464. doi: 10.7554/eLife.67464 (PMC9307272; doi:10.7554/eLife.67464)
Supplement: Supplementary file 3. — SE-B, SE-Y, standard error of B and Y-intercept; Y-int., Y-intercept (CT value); R2, squared correlation coefficient; LOD, limit of detection (number of transcripts); LOQ, limit of quantification (number of transcripts); E (%), Efficiency in % (E=10–1/slope-1), 100% efficiency corresponds to a slope of –3.32. [file elife-67464-supp3.docx]

**Supplemetary File 3**

| **β-subunit** | **B** | **SE-B** | **Y-int.** | **SE-Y** | **R^2^** | **LOD** | **LOQ** | **E (%)** |
| --- | --- | --- | --- | --- | --- | --- | --- | --- |
| β1 | -3.246 | 0.009 | 35.15 | 0.13 | 0.997 | 5 | 100 | 103 |
| β2 | -3.382 | 0.024 | 37.81 | 0.24 | 0.996 | 5 | 30 | 98 |
| β3 | -3.263 | 0.032 | 35.30 | 0.16 | 0.995 | 5 | 50 | 103 |
| β4 | -3.231 | 0.032 | 36.48 | 0.38 | 0.994 | 10 | 100 | 104 |
| β2a | -3.283 | 0.037 | 35.04 | 0.54 | 0.981 | 10 | 100 | 101 |
| β2b | -3.334 | 0.070 | 34.36 | 0.14 | 0.990 | 10 | 120 | 99 |
| β2c+d | -3.160 | 0.023 | 35.78 | 0.02 | 0.998 | 5 | 20 | 107 |
| β2e | -3.070 | 0.005 | 35.30 | 0.12 | 0.993 | 5 | 50 | 111 |
