# Supplementary material for: β2-subunit alternative splicing stabilizes Cav2.3 Ca2+ channel activity during continuous midbrain dopamine neuron-like activity
Source: eLife. 2022 Jul 6;11:e67464. doi: 10.7554/eLife.67464 (PMC9307272; doi:10.7554/eLife.67464)
Supplement: Supplementary file 4. — Data and statistics of genes as indicated for graphs shown in Figure 2—figure supplement 2A (middle) and B (right). n represents number of analyzed dopaminergic neurons derived from N individual mice. Significances according to two-way ANOVA followed by Tukey’s multiple comparisons test: ***, P<0.001. [file elife-67464-supp4.docx]

**Supplemetary File 4**

|  | **A. mRNA expression in SN DA neurons of juvenile wildtype mice** | | | | | | | | | | | |  |
| --- | --- | --- | --- | --- | --- | --- | --- | --- | --- | --- | --- | --- | --- |
| **analyzed mRNA** | **mean** | **±SD** | **±SEM** | | **median** | | | **n/N** | | | | |  |
| **β2** | 0.15 | 0.13 | 0.04 | | 0.16 | | | 10/2 | | | | |  |
|  | | | | | | | | | | | | |  |
|  | **B. number of mRNA molecules in DA neurons of adult wildtype mice** | | | | | | | | | | | |  |
|  | **SN** | | | | | | **VTA** | | | | | |  |
| **analyzed mRNA** | **mean** | **SD** | **SEM** | **median** | | **N** | **mean** | | **SD** | **SEM** | **median** | **N** | |
| **β2a** | 4.72 | 0.40 | 0.16 | 4.86 | | 6 | 4.62 | | 0.42 | 0.17 | 4.67 | 6 | |
| **β2e** | 9.68*** | 1.76 | 0.72 | 10.25 | | 6 | 8.43*** | | 1.93 | 0.79 | 8.13 | 6 | |
