# Supplementary material for: β2-subunit alternative splicing stabilizes Cav2.3 Ca2+ channel activity during continuous midbrain dopamine neuron-like activity
Source: eLife. 2022 Jul 6;11:e67464. doi: 10.7554/eLife.67464 (PMC9307272; doi:10.7554/eLife.67464)
Supplement: Supplementary file 5. — F: forward primer, R: reverse primer [file elife-67464-supp5.docx]

**Supplemetary File 5**

| **Gene** | **Primer** | **Sequence (5`-3`)** | **Genbank accession no. (NCBI)** | **5`-Position** | **Amplicon size [bp]** |
| --- | --- | --- | --- | --- | --- |
| Mouse  Calbindin-d28k  (CB) | F (outer) | CGCACTCTCAAACTAGCCG | M21531 | 87 | 891 |
|  | R (outer) | CAGCCTACTTCTTTATAGCGCA |  | 977 |  |
|  | F (inner) | GAGATCTGGCTTCATTTCGAC |  | 167 | 440 |
|  | R (inner) | AGTTCCAGCTTTCCGTCATTA |  | 606 |  |
| Mouse  Glial fibrillary acidic protein (GFAP) | F (outer) | AGAACAACCTGGCTGCGTAT | K01347 | 407 | 786 |
|  | R (outer) | GCTCCTGCTTCGAGTCCTTA |  | 1192 |  |
|  | F (inner) | AGAAAGGTTGAATCGCTGGA |  | 472 | 517 |
|  | R (inner) | CCAGGGCTAGCTTAACGTTG |  | 988 |  |
| Mouse Glutamate decarboxylase (GAD65) | F (outer) | CATACGCAGACAGCACGTTT | NM_008078.1 | 166 | 905 |
|  | R (outer) | AAAAGATTCCATCGCCAGAG |  | 1070 |  |
|  | F (inner) | GGGATGTCAACTACGCGTTT |  | 606 | 389 |
|  | R (inner) | CACAAATACAGGGGCGATCT |  | 994 |  |
| Mouse Glutamate decarboxylase (GAD67) | F (outer) | TGACATCGACTGCCAATACC | Z49976 | 731 | 1105 |
|  | R (outer) | GGGTTAGAGATGACCATCCG |  | 1835 |  |
|  | F (inner) | CATATGAAATTGCACCCGTG |  | 761 | 702 |
|  | R (inner) | CGGTGTCATAGGAGACGTCA |  | 1462 |  |
| Mouse Tyrosine hydroxylase  (TH) | F (outer) | CACCTGGAGTACTTTGTGCG | M69200 | 387 | 1139 |
|  | R (outer) | CCTGTGGGTGGTACCCTATG |  | 1525 |  |
|  | F (inner) | TGCACACAGTACATCCGTCA |  | 936 | 377 |
|  | R (inner) | TCTGACACGAAGTACACCGG |  | 1312 |  |
| Mouse Cav2.3  R-type calcium channel (Cacna1e) | F (outer II-III loop) | CCATCTACTTCATTGTGCTCACC | NM_009782.3 | 2043 | 816* |
|  | R (outer II-III loop) | CCCCTTCATCCAGACTCCG |  | 2858 |  |
|  | F (outer C-terminus) | GACAGCAGCTGGAAGAACAGA |  | 5670 | 641* |
|  | R (outer C-terminus) | GATGGAGCTCTCACTTAGGGAAC |  | 6310 |  |
|  | F (inner II-II loop) | GGAGGTCAGCCCGATGTC |  | 2203 | 420* |
|  | R (inner II-III loop) | GGGCTCCTCTGGTTGTCC |  | 2622 |  |
|  | F (inner C-terminus) | CTGAGTGGTCGGAGTGGATAC |  | 5795 | 369* |
|  | F (innerC-terminus) | AGAGAGGAGGTGCTTTCGTTC |  | 6163 |  |
