# Supplementary material for: β2-subunit alternative splicing stabilizes Cav2.3 Ca2+ channel activity during continuous midbrain dopamine neuron-like activity
Source: eLife. 2022 Jul 6;11:e67464. doi: 10.7554/eLife.67464 (PMC9307272; doi:10.7554/eLife.67464)
Supplement: Supplementary file 6. — All values are given as mean ± SEM for the indicated number of experiments (n, N=2). Parameters were obtained as described in Materials and Methods from a holding potential of –89 mV using 15 mM Ca2+ as the charge carrier. Voltage-dependence of gating: Parameters are as given in legend to Table 1. Statistical significance was determined using one-way ANOVA with Bonferroni post-hoc test (V0.5, Vrev, act thresh, V0.5,inact, kinact, plateau) or Kruskal-Wallis followed by Dunn’s multiple comparison test (k). Statistical significances of post hoc tests are indicated for comparison vs. β2a (*, **, ***) or vs. β3 (§, §§, §§§): *** P<0.001; ** P<0.01; * P<0.05. Inactivation time course: The r values represent the fraction of ICa remaining after 50, 100, 250, 500, 1,000 or 5000 ms during a 5 s pulse to Vmax. Statistical significance was determined using one-way ANOVA with Bonferroni post-hoc test. Statistical significances of post hoc tests are indicated for comparison vs. β2a: *** P<0.001; ** P<0.01; * P<0.05. [file elife-67464-supp6.docx]

**Supplemetary File 6**

|  | **Cav1.3_L_ - Activation 15 mM Ca^2+^** | | | | | | | | | | **Cav1.3_L_ - Inactivation 15 mM Ca^2+^** | | | | | | | | | |
| --- | --- | --- | --- | --- | --- | --- | --- | --- | --- | --- | --- | --- | --- | --- | --- | --- | --- | --- | --- | --- |
| **β-subunit** | **V_0.5_**  **[mV]** | | **k**  **[mV]** | **V_rev_**  **[mV]** | | **act**  **thresh**  **[mV]** | **current**  **density**  **[pA/pF]** | | **n** | | **V_0.5, inact_**  **[mV]** | | | **k_inact_**  **[mV]** | | | | **plateau**  **[%]** | | **n** |
| β2a | 4.3  ±1.8 | | 9.9  ±0.3 | 71.2  ±2.0 | | -34.5  ±0.9 | -7.8  ±0.6 | | 14 | | -18.3  ±3.2 | | | 10.0  ±1.0 | | | 36.2  ±5.8 | | | 5 |
| _C3S/C4S_β2a | 0.2  ±1.2 | | 9.2*  ±0.2 | 71.3  ±1.1 | | -34.9^§§^  ±0.9 | -10.9  ±1.6 | | 15 | | -24.4  ±1.2 | | | 6.1***  ±0.4 | | | 21.6*  ±1.9 | | | 11 |
| β3 | 3.1  ±1.9 | | 8.9*  ±0.4 | 71.1  ±2.7 | | -30.6*  ±0.8 | -10.2  ±1.4 | | 9 | | -20.3  ±0.9 | | | 7.0*  ±0.5 | | | 28.5  ±5.4 | | | 5 |
| **Cav1.3_L_ - 5 s Inactivation 15 mM Ca^2+^** | | | | | | | | | | | | | | | | | | | | |
| **β-subunit** | | **r50 [%]** | | | **r100 [%]** | | | **r250 [%]** | | **r500 [%]** | | | **r1000 [%]** | | | **r5000 [%]** | | | **n** | |
| β2a | | 86.8  ±2.7 | | | 81.2  ±3.0 | | | 71.5  ±3.5 | | 63.3  ±4.1 | | 54.6  ±4.2 | | | 36.0  ±3.7 | | | | 10 | |
| _C3S/C4S_β2a | | 74.5*  ±3.0 | | | 62.7***  ±2.6 | | | 47.1***  ±3.2 | | 33.0***  ±2.6 | | 23.3***  ±2.6 | | | 12.6***  ±1.7 | | | | 14 | |
| β3 | | 81.5  ±3.4 | | | 70.3  ±3.7 | | | 50.6**  ±4.1 | | 34.6***  ±3.9 | | 25.2***  ±4.2 | | | 13.7***  ±3.0 | | | | 7 | |
