# Supplementary material for: β2-subunit alternative splicing stabilizes Cav2.3 Ca2+ channel activity during continuous midbrain dopamine neuron-like activity
Source: eLife. 2022 Jul 6;11:e67464. doi: 10.7554/eLife.67464 (PMC9307272; doi:10.7554/eLife.67464)
Supplement: Supplementary file 7. — For details see results. Experiments were performed as described in detail in the Methods section and Figure 6—figure supplement 1. [file elife-67464-supp7.docx]

**Supplemetary File 7**

| **Effect of SNX-482 (100 nM) on *I*_Ca_ peak amplitude (pA)** | | | | | |
| --- | --- | --- | --- | --- | --- |
|  | mean | SD | SEM | median | n/N |
| Control | 1220 | 372 | 131 | 1170 | 8/7 |
| SNX-482 | 1070 | 342 | 121 | 1110 | 8/7 |
| p-value | 0.0276 (two-tailed paired t-test) | | | | |
| **Remaining *I*_Ca_ peak amplitude in SNX-482 (%)** | | | | | |
| Control vs. SNX-482 | 87 | 11 | 4 | 90 | 8/7 |
| p-value | 0.0122 (two-tailed one sample t-test) | | | | |
| **Effect of nifedipine (10 µM) on *I*_Ca_ peak amplitude (pA)** | | | | | |
|  | mean | ±SD | ±SEM | median | n |
| Control | 1240 | 351 | 143 | 1240 | 6/6 |
| Nifedipine | 1120 | 312 | 127 | 1120 | 6/6 |
| p-value | 0.0312 (two-tailed Wilcoxon test) | | | | |
| **Remaining *I*_Ca_ peak amplitude in nifedipine (%)** | | | | | |
| Control vs. nifedipine | 90 | 3 | 1.4 | 91 | 6/6 |
| p-value | 0.0312 (two-tailed Wilcoxon signed rank test) | | | | |
