# Supplementary figures and images for: β2-subunit alternative splicing stabilizes Cav2.3 Ca2+ channel activity during continuous midbrain dopamine neuron-like activity
Source: eLife. 2022 Jul 6;11:e67464. doi: 10.7554/eLife.67464 (PMC9307272; doi:10.7554/eLife.67464)

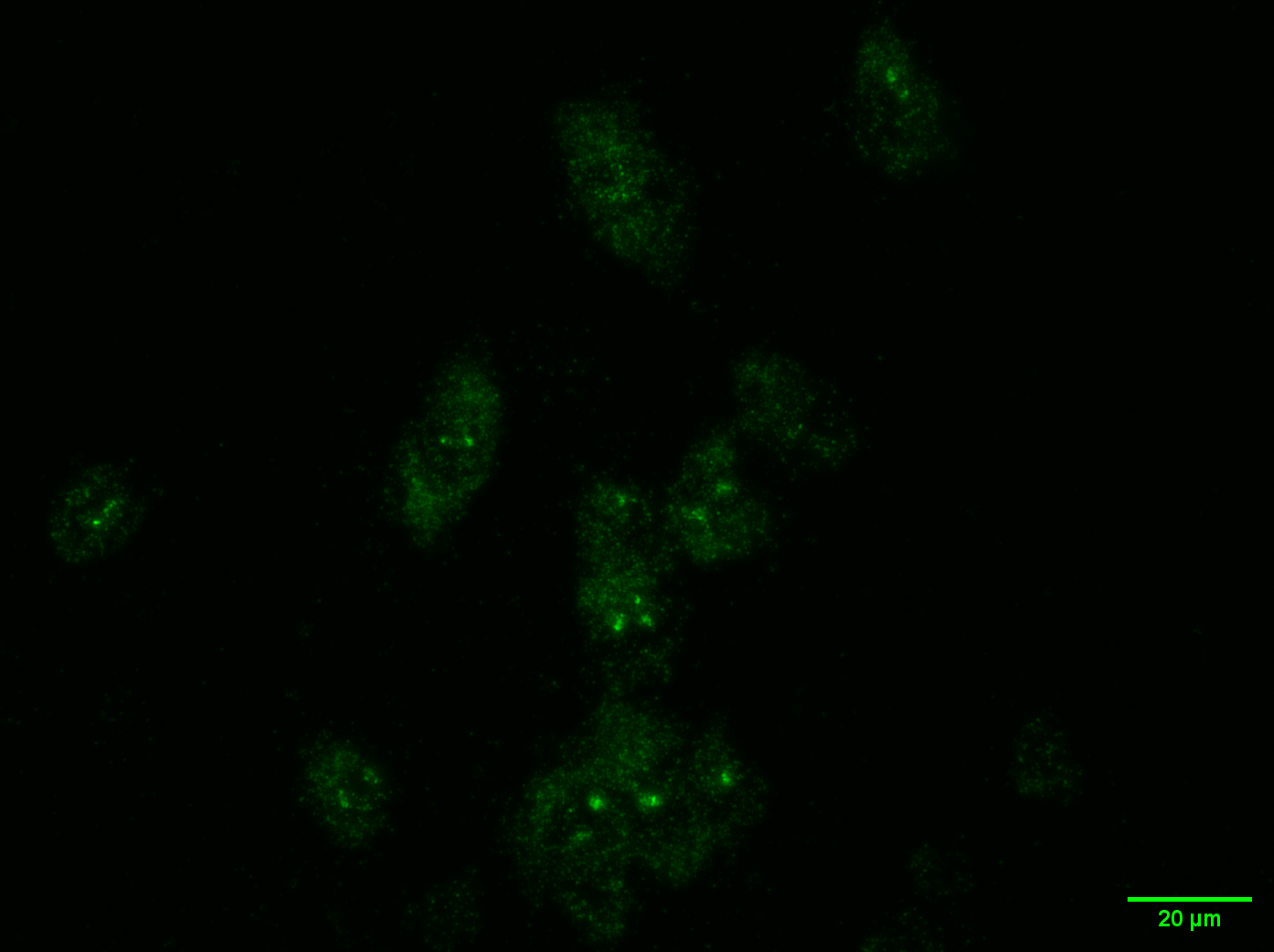

Supplement: Source data 1. [file elife-67464-data1.zip › Siller_et_al_2022/Figure_2-figure supplement 2_B_THu_original.png]

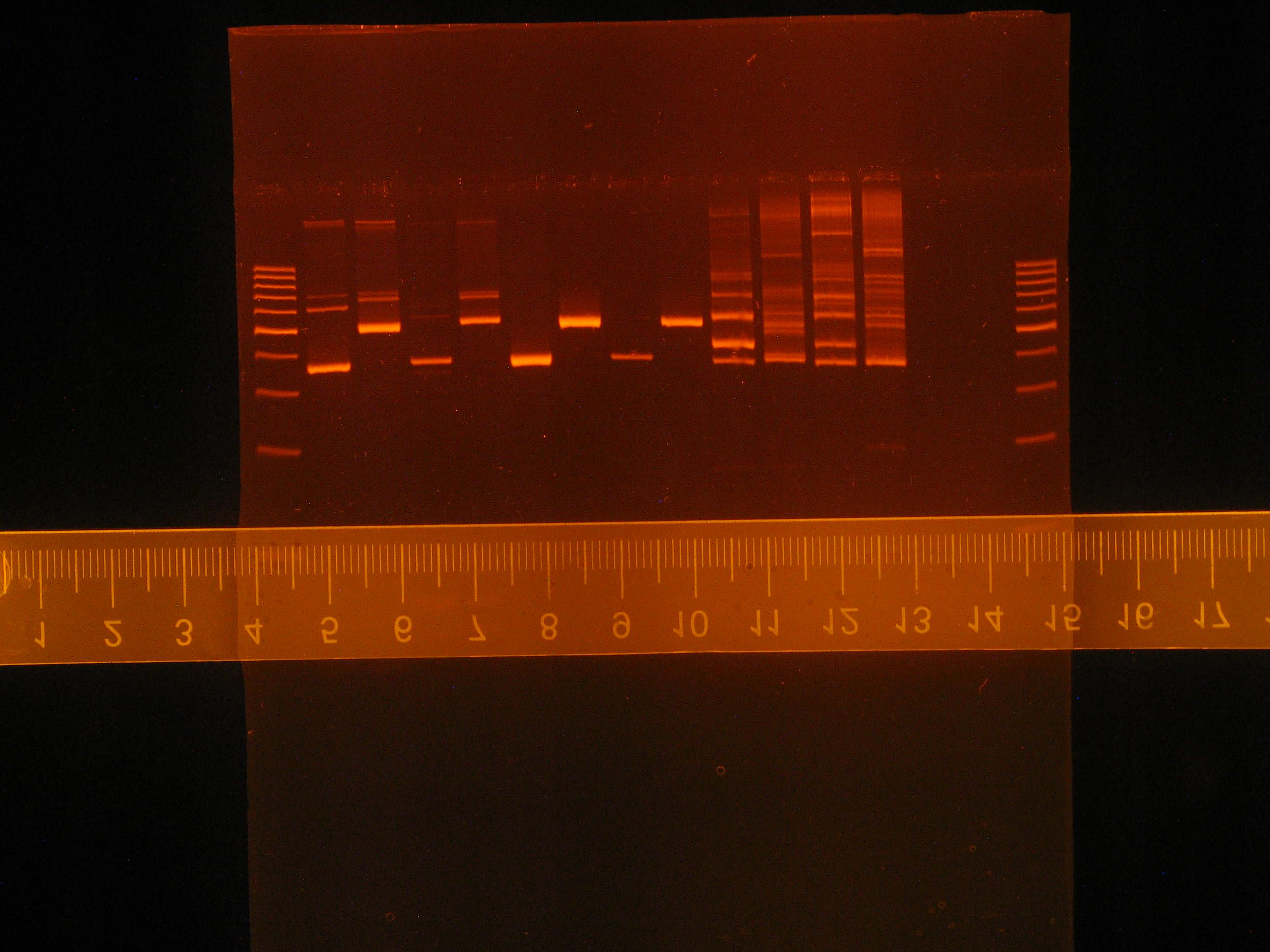

Supplement: Source data 1. [file elife-67464-data1.zip › Siller_et_al_2022/Figure_1-figure supplement 1_Bl_original.jpg]

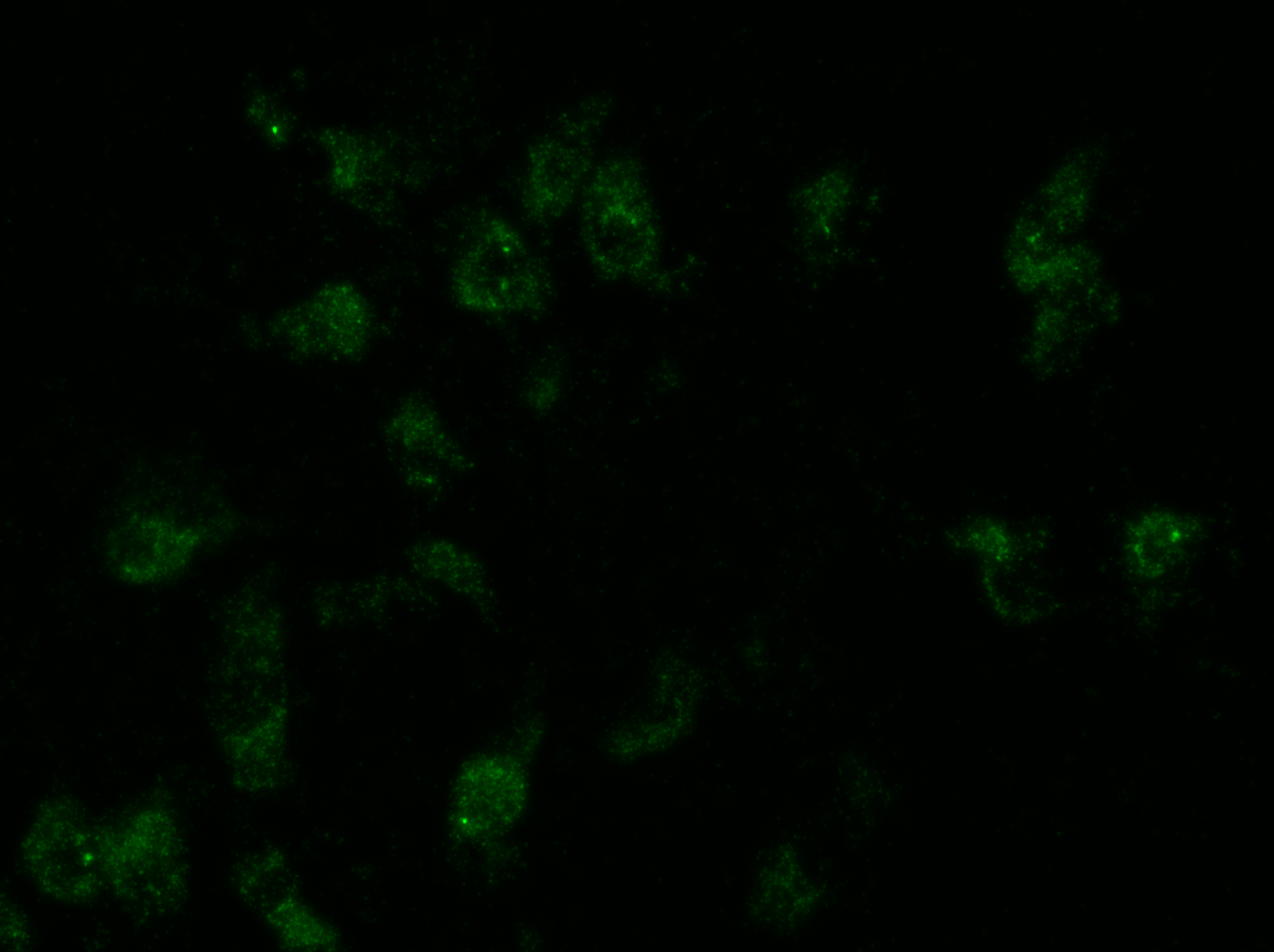

Supplement: Source data 1. [file elife-67464-data1.zip › Siller_et_al_2022/Figure_2-figure supplement 2_B_THl_original.png]

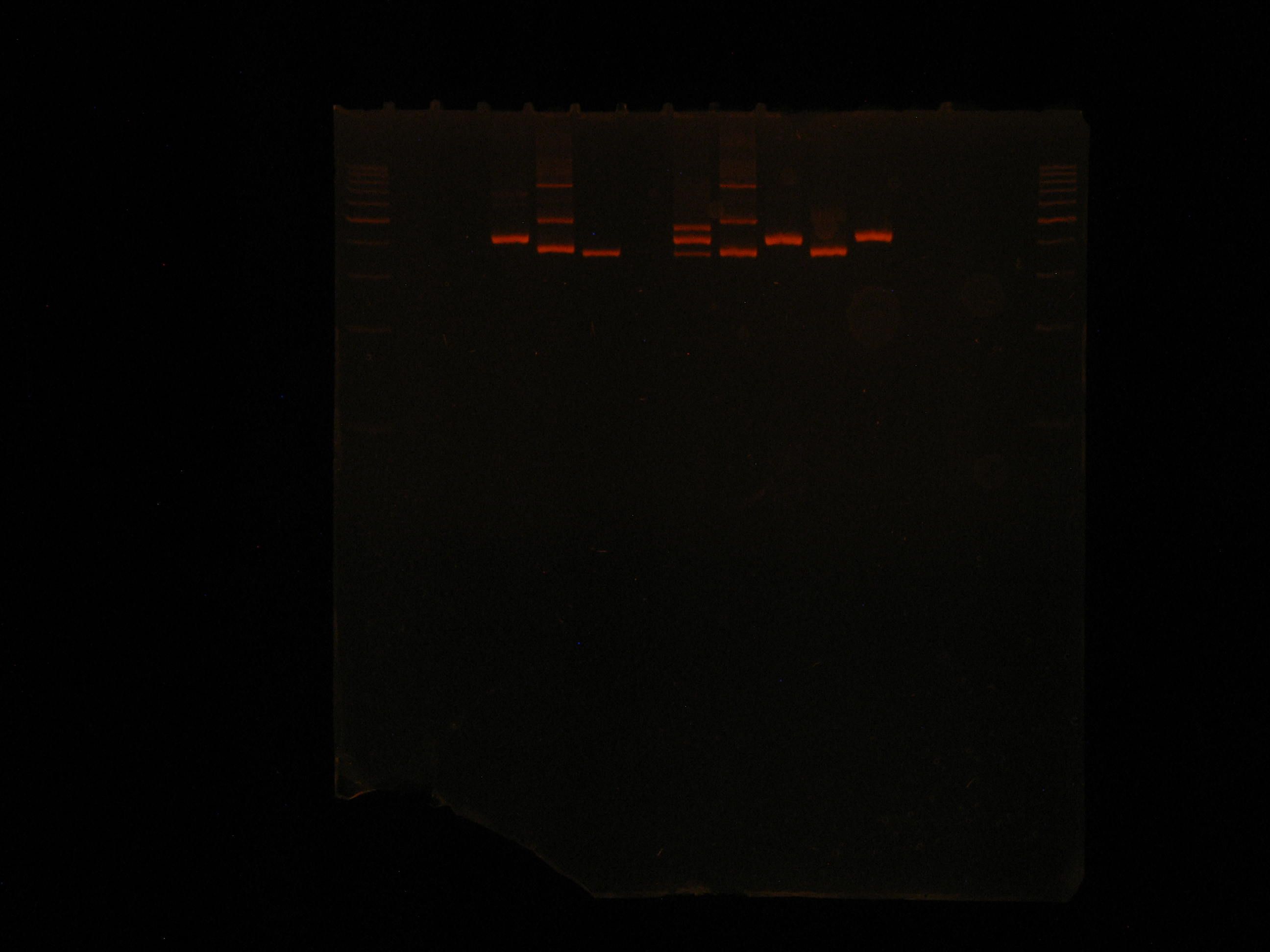

Supplement: Source data 1. [file elife-67464-data1.zip › Siller_et_al_2022/Figure_1-figure supplement 1_Br_original.jpg]

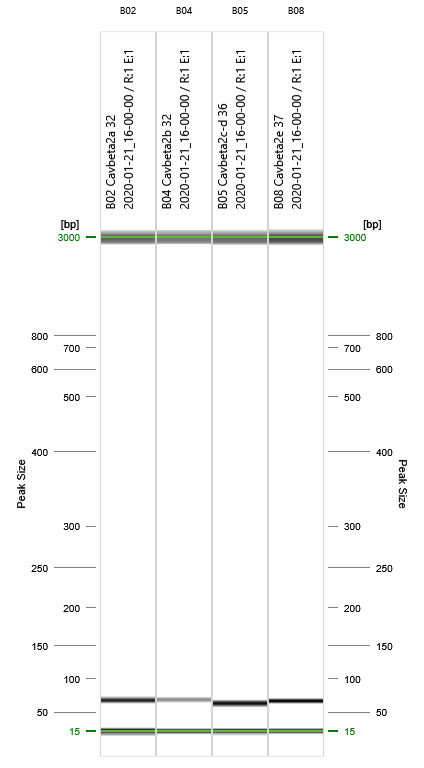

Supplement: Source data 1. [file elife-67464-data1.zip › Siller_et_al_2022/Figure_2-figure supplement 2_Aur_original.Png]

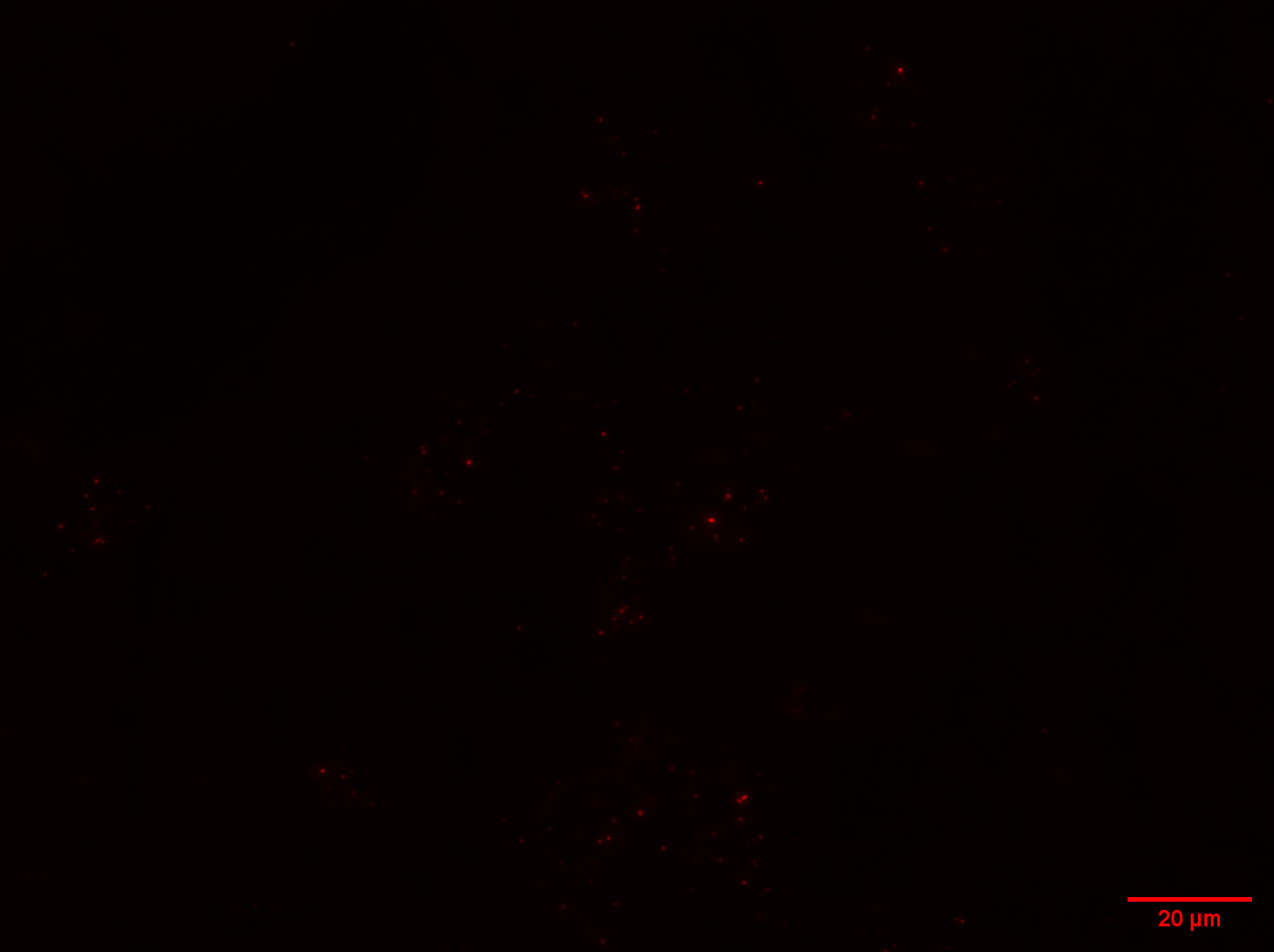

Supplement: Source data 1. [file elife-67464-data1.zip › Siller_et_al_2022/Figure_2-figure supplement 2_B_beta2a_original.png]

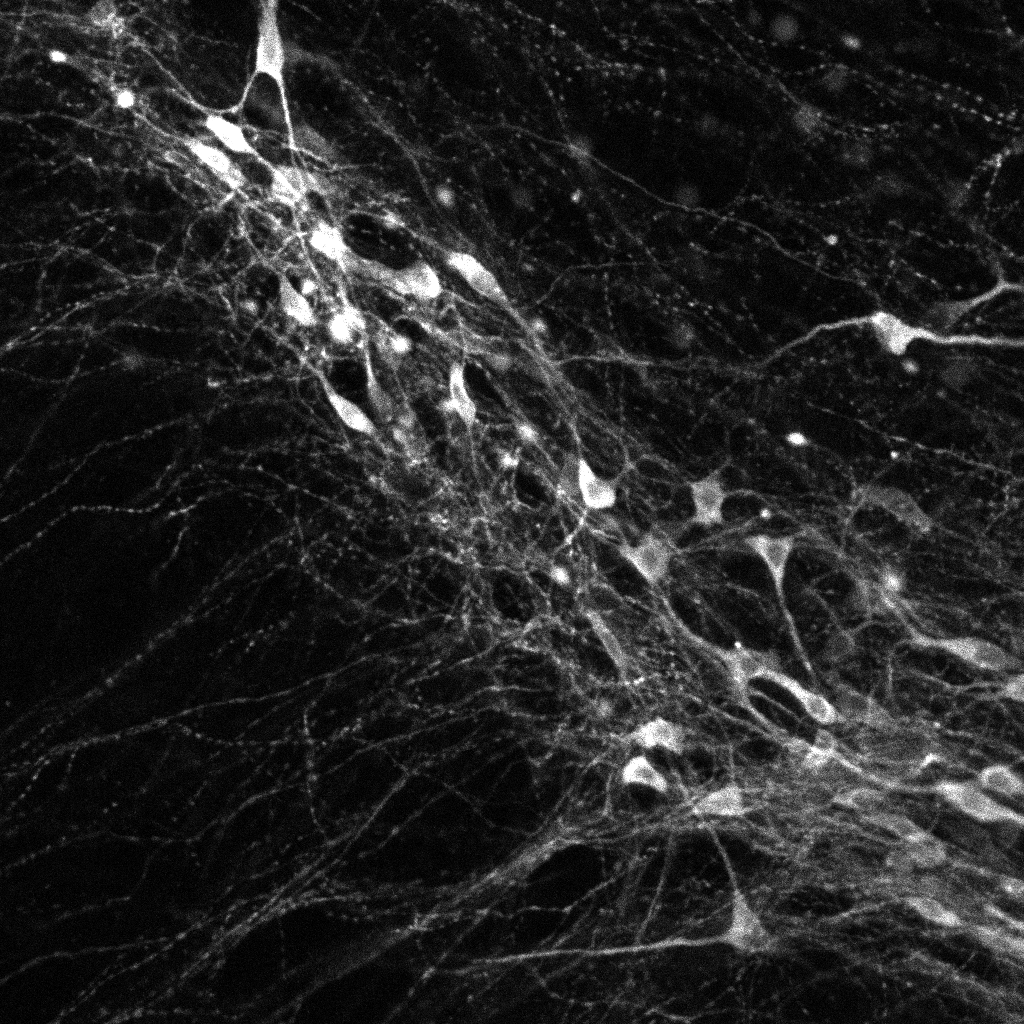

Supplement: Source data 1. [file elife-67464-data1.zip › Siller_et_al_2022/Figure_6_figure_supplement_1_J_2.png]

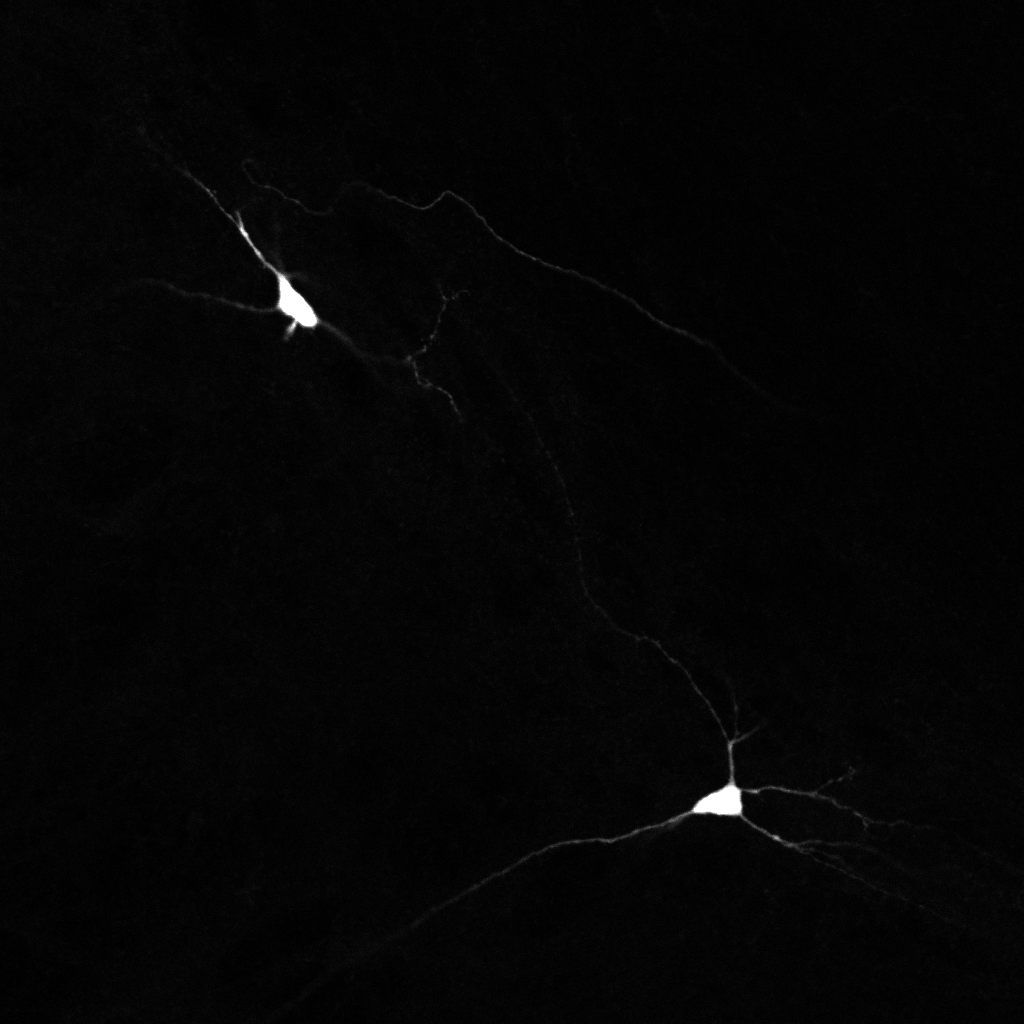

Supplement: Source data 1. [file elife-67464-data1.zip › Siller_et_al_2022/Figure_6_figure_supplement_1_J_1.png]

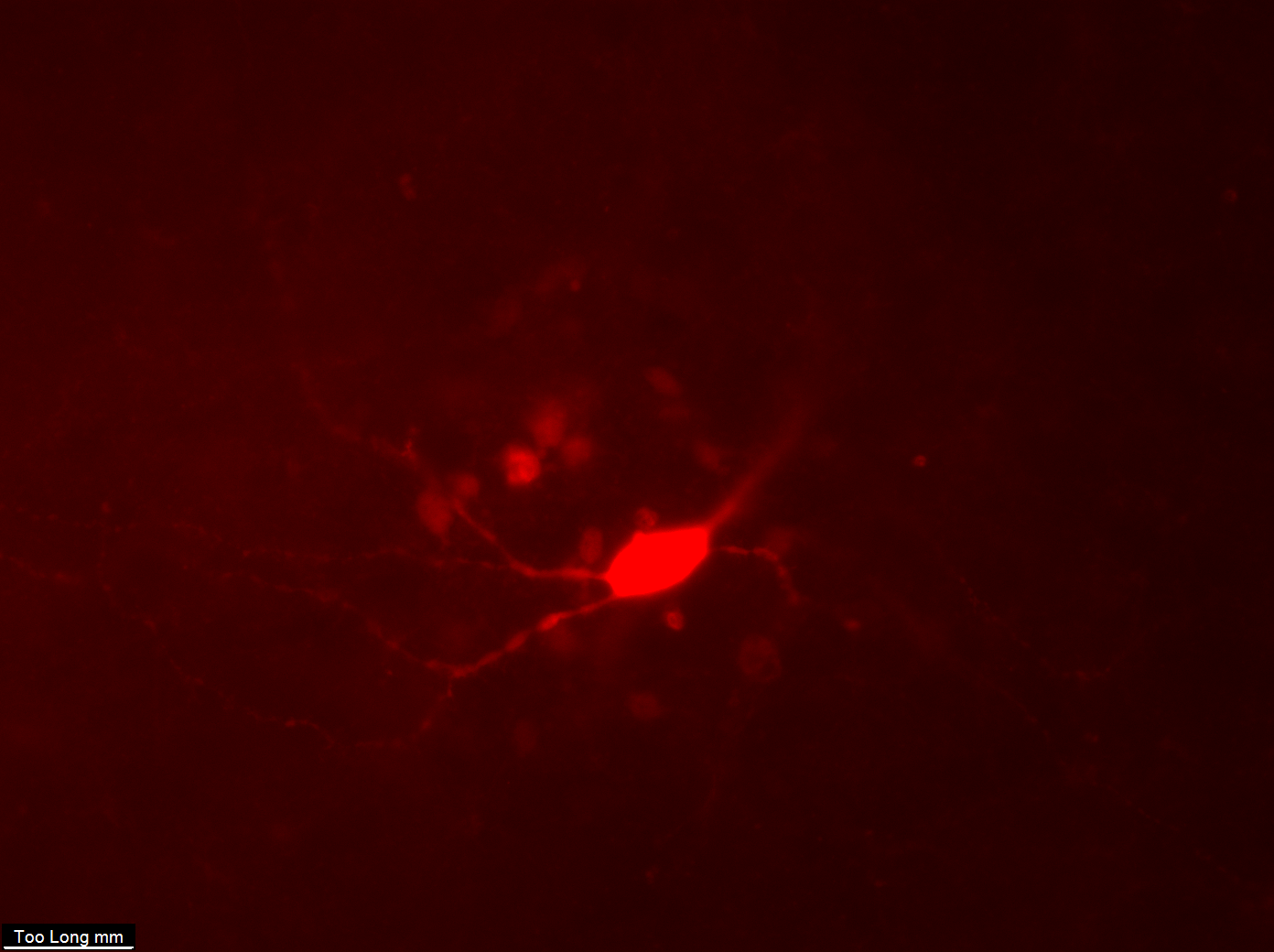

Supplement: Source data 1. [file elife-67464-data1.zip › Siller_et_al_2022/Figure_6_C_NB_original.png]

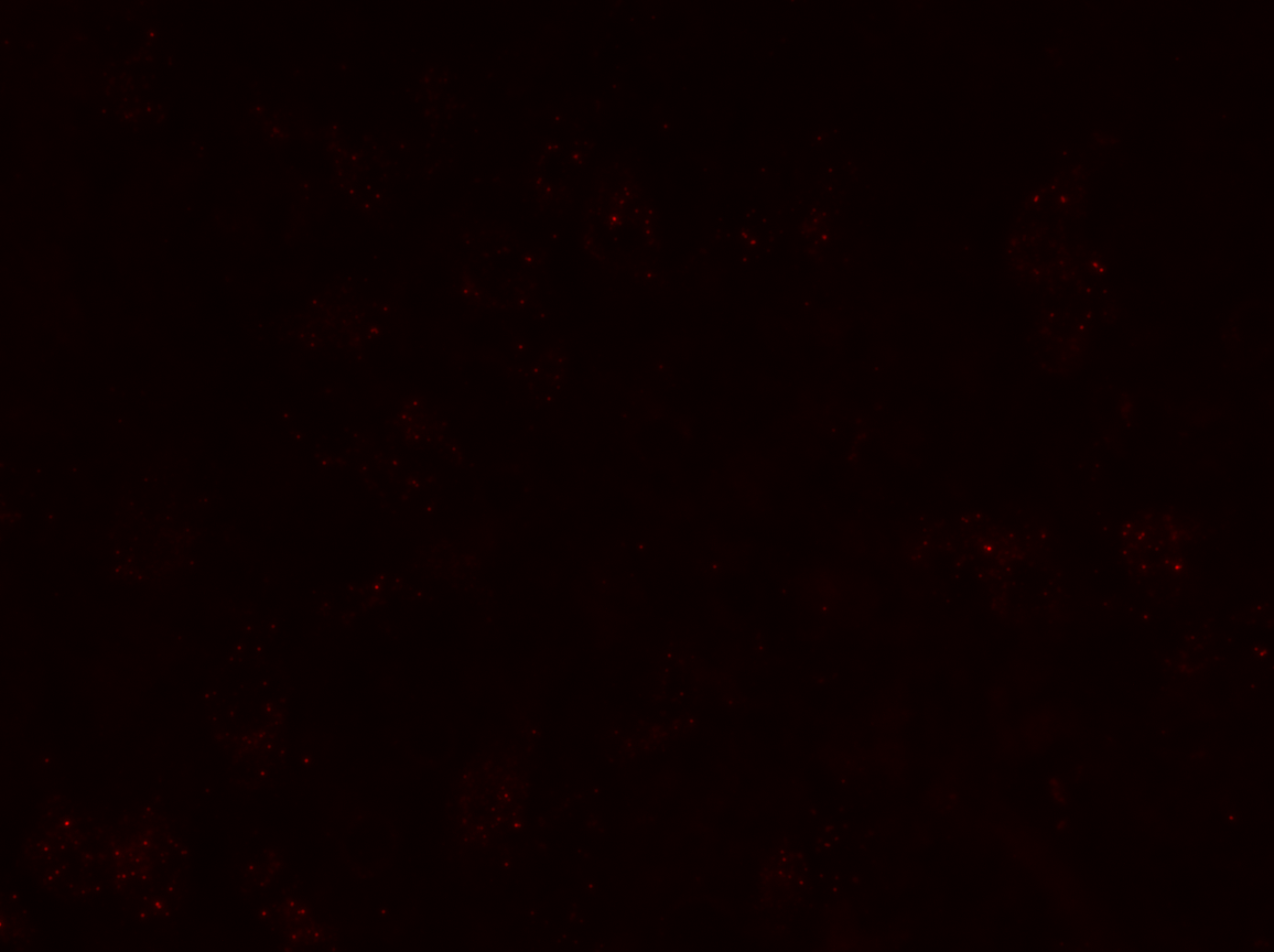

Supplement: Source data 1. [file elife-67464-data1.zip › Siller_et_al_2022/Figure_2-figure supplement 2_B_beta2e_original.png]

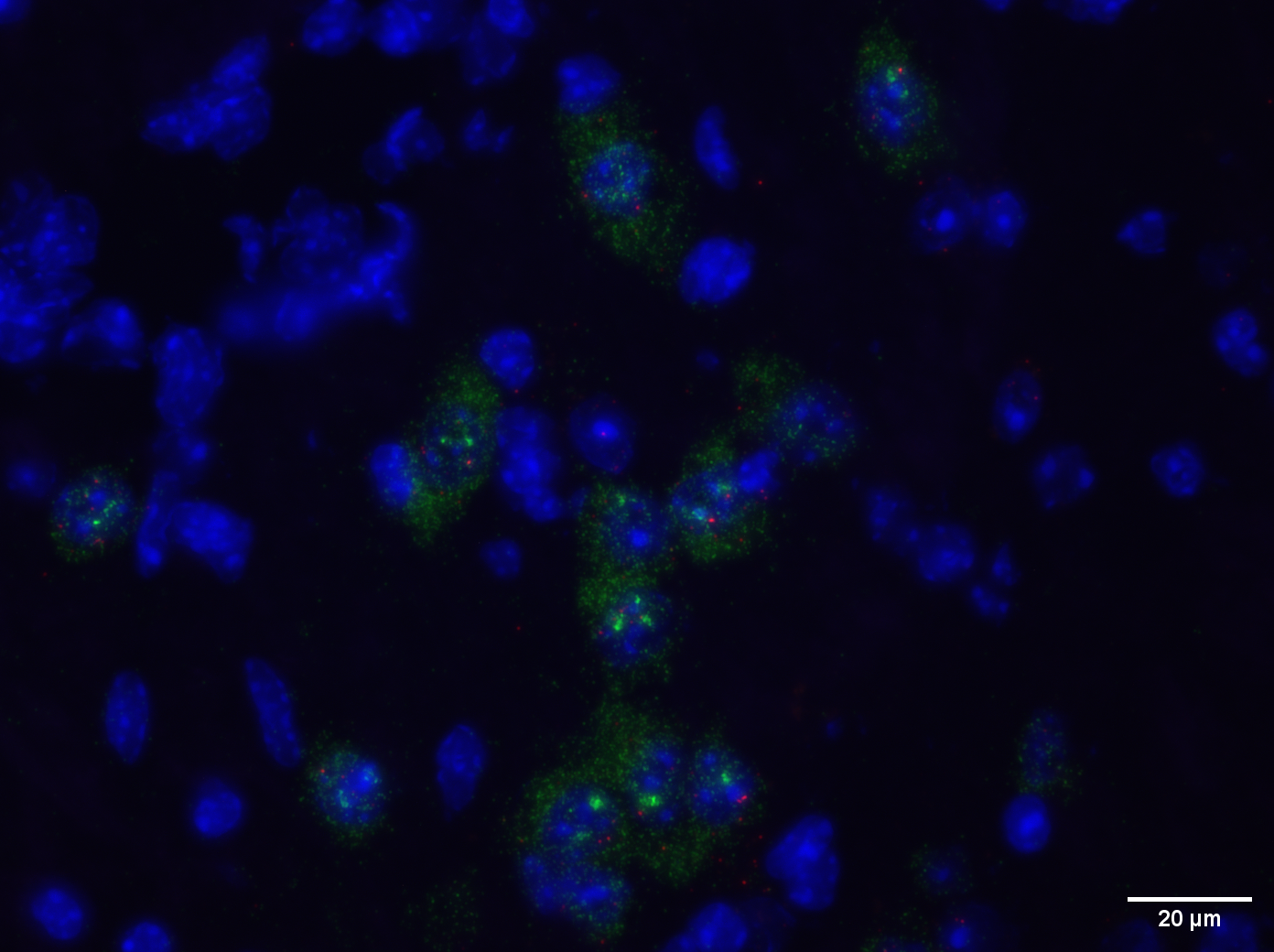

Supplement: Source data 1. [file elife-67464-data1.zip › Siller_et_al_2022/Figure_2-figure supplement 2_B_THu_beta2a_merge_original.png]

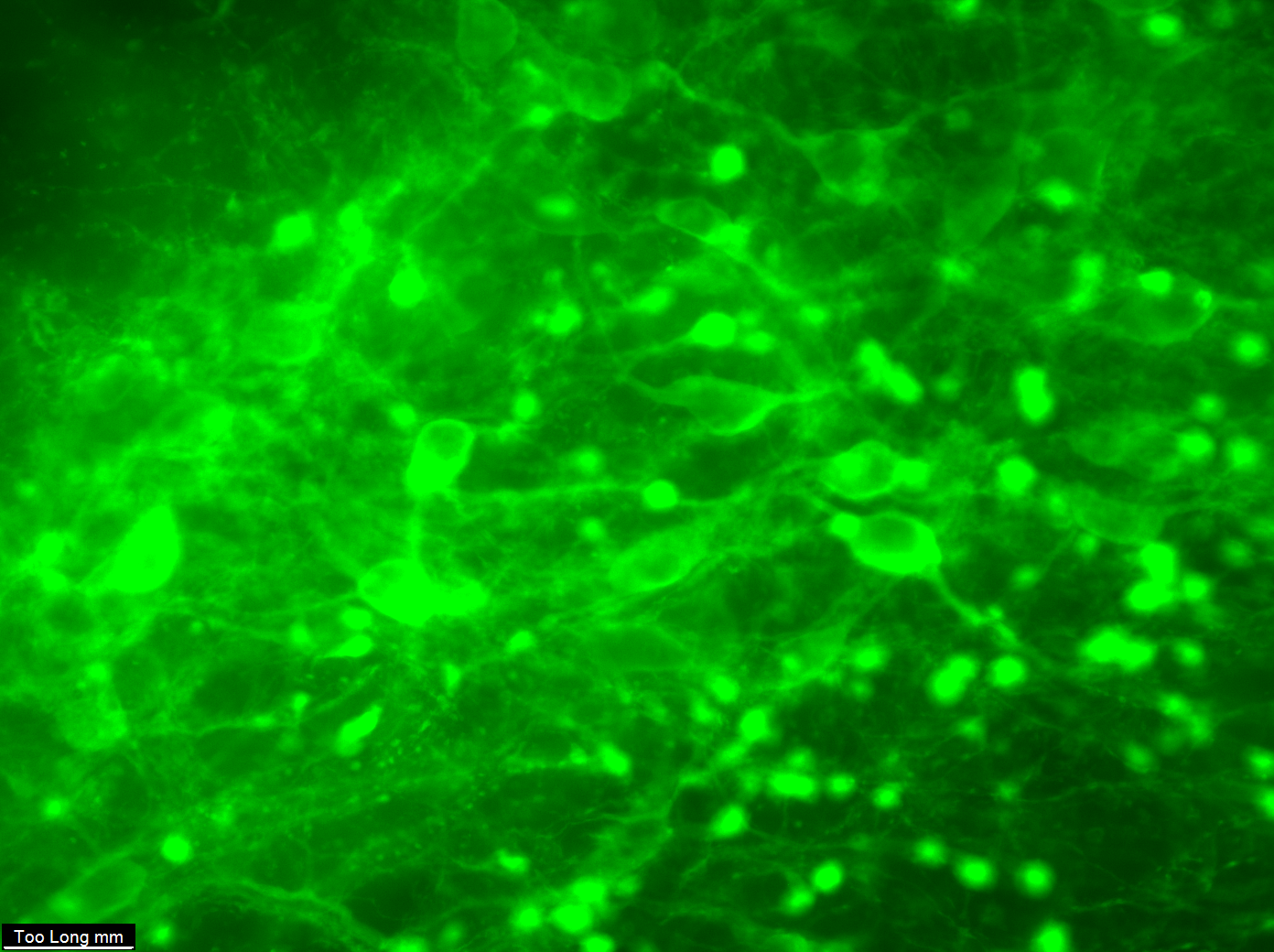

Supplement: Source data 1. [file elife-67464-data1.zip › Siller_et_al_2022/Figure_6_C_TH_original.png]

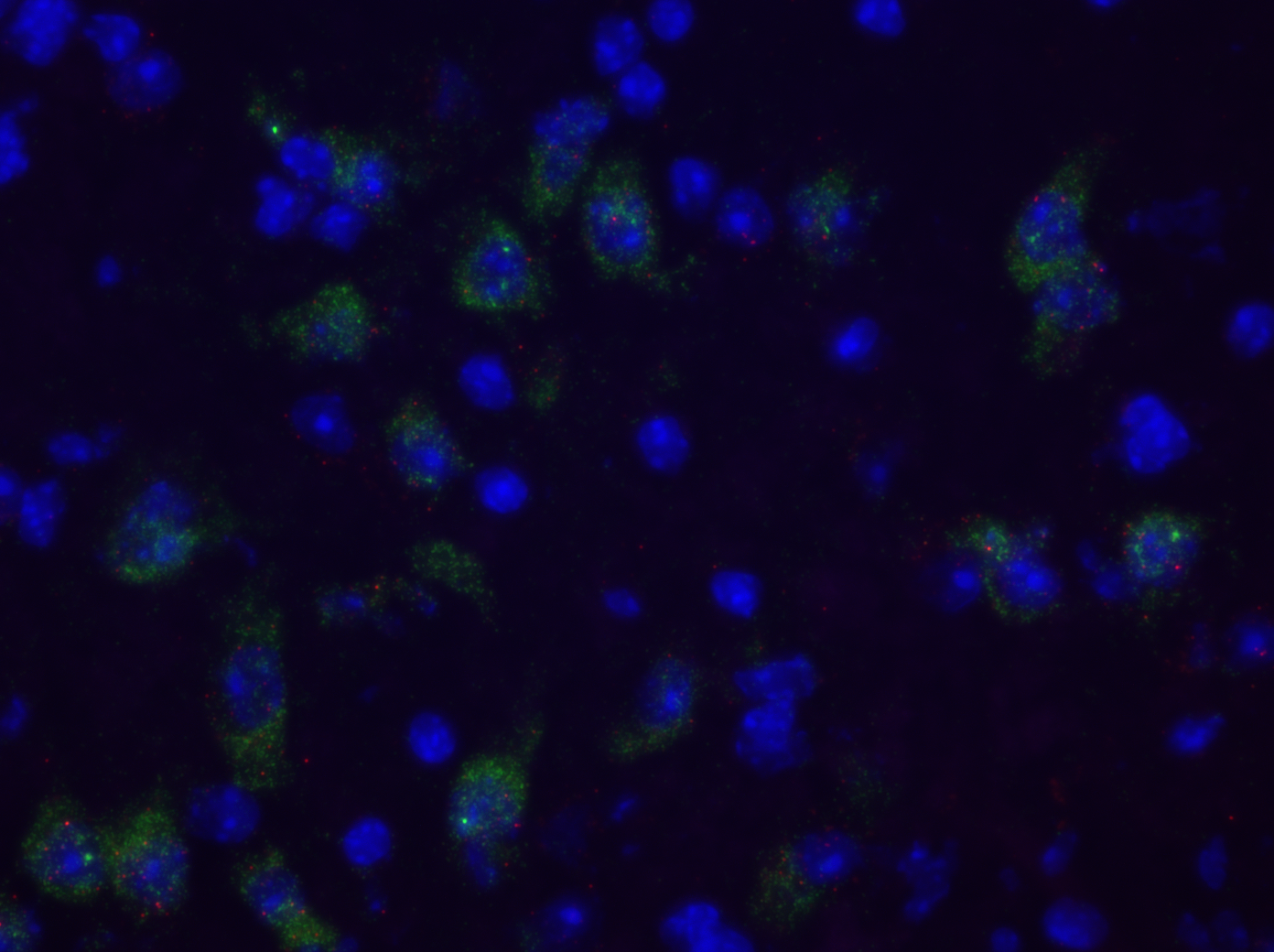

Supplement: Source data 1. [file elife-67464-data1.zip › Siller_et_al_2022/Figure_2-figure supplement 2_B_THl_beta2e_merge_original.png]

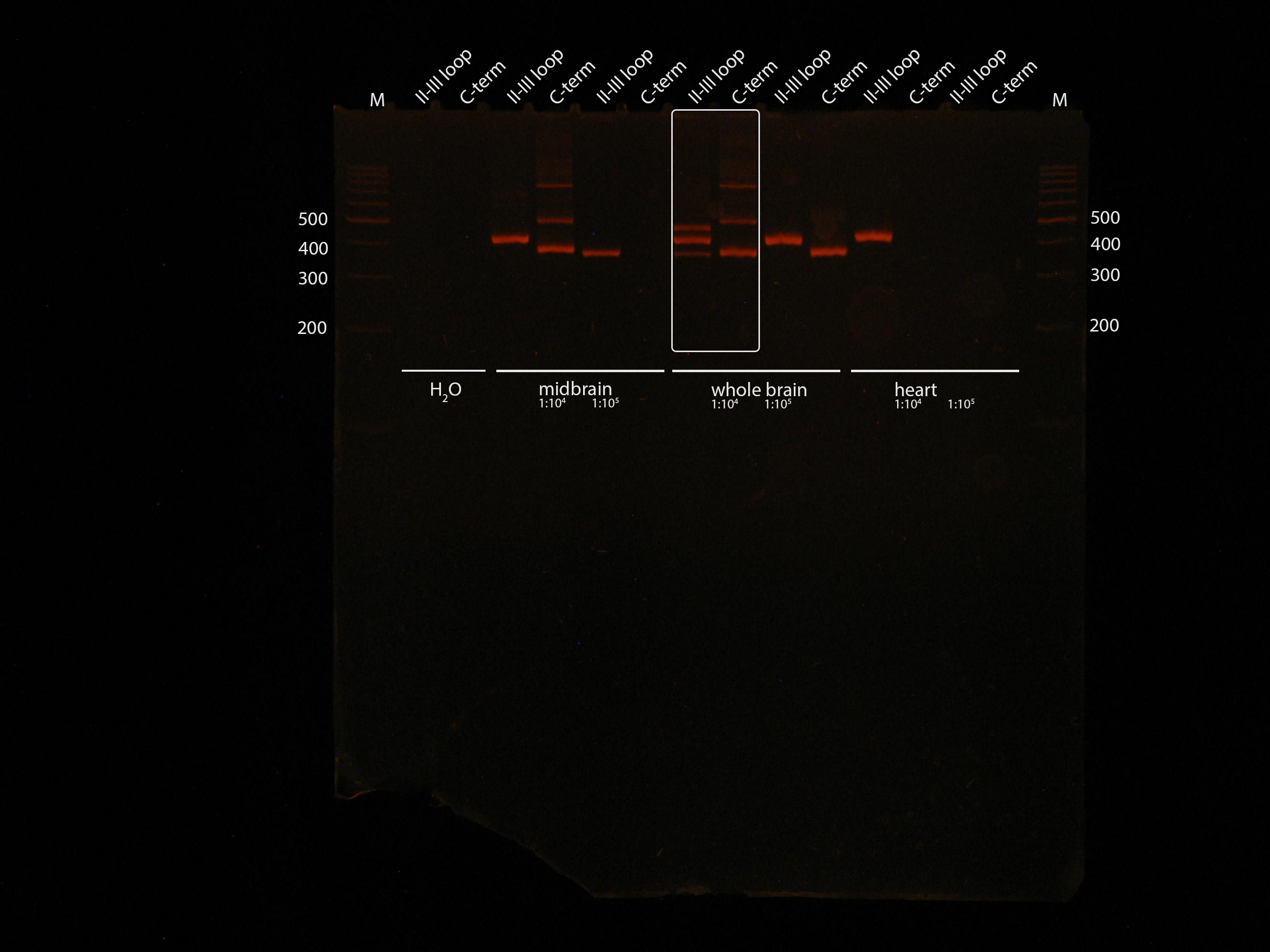

Supplement: Source data 1. [file elife-67464-data1.zip › Siller_et_al_2022/Figure_1-figure supplement 1_Br_original-labelled.tif]

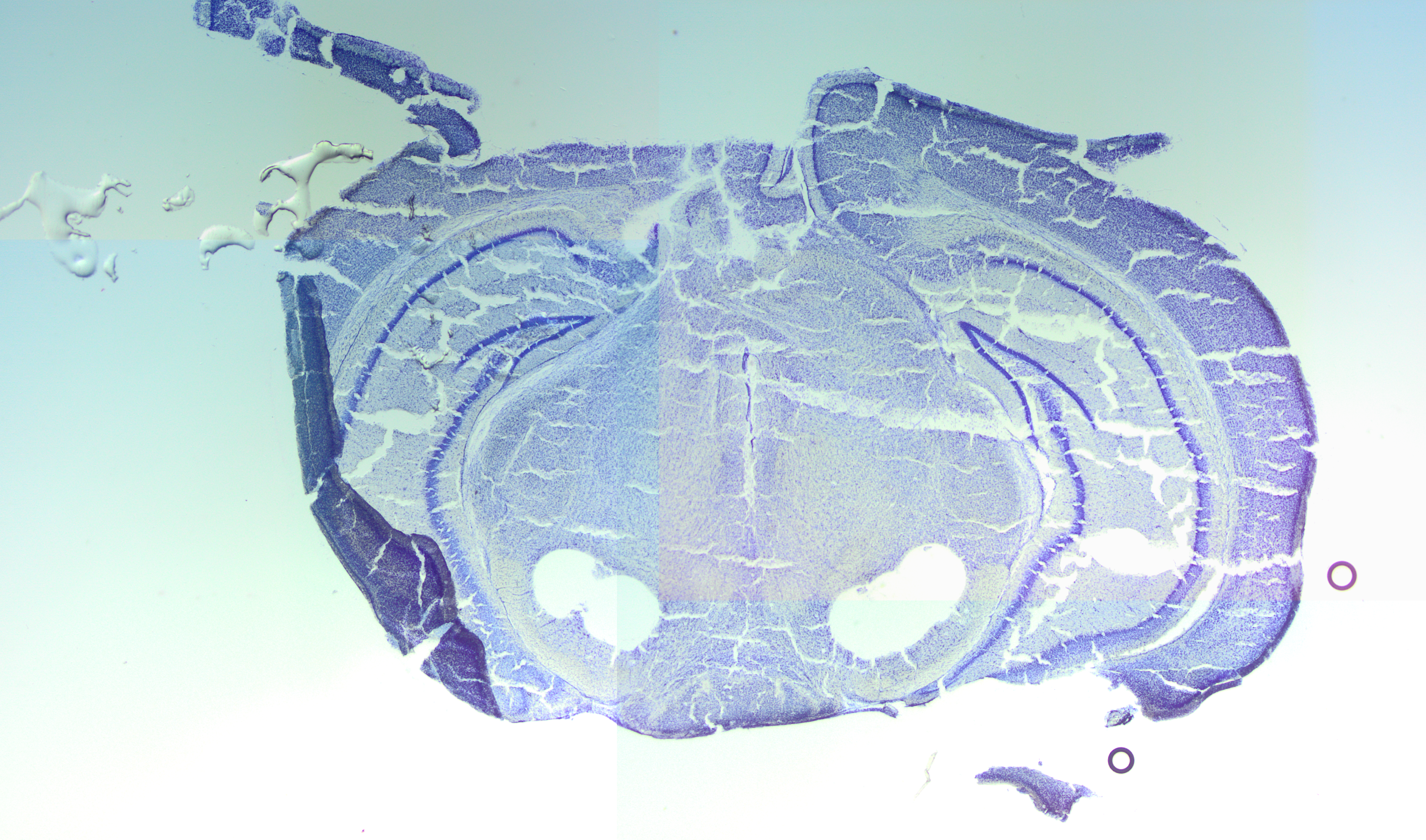

Supplement: Source data 1. [file elife-67464-data1.zip › Siller_et_al_2022/Figure_2_Cl_original.tif]

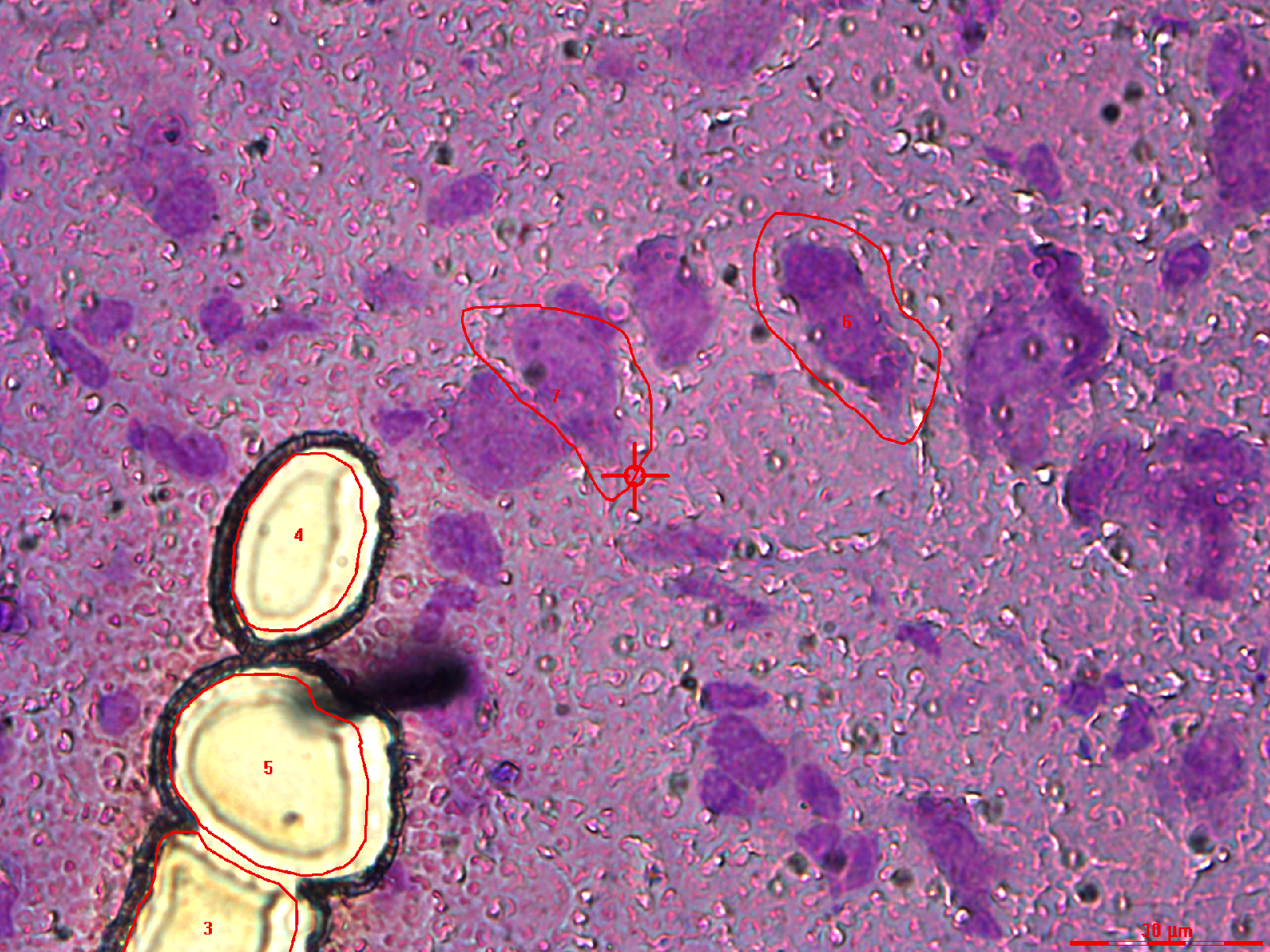

Supplement: Source data 1. [file elife-67464-data1.zip › Siller_et_al_2022/Figure_2-figure supplement 2_All_original.tif]

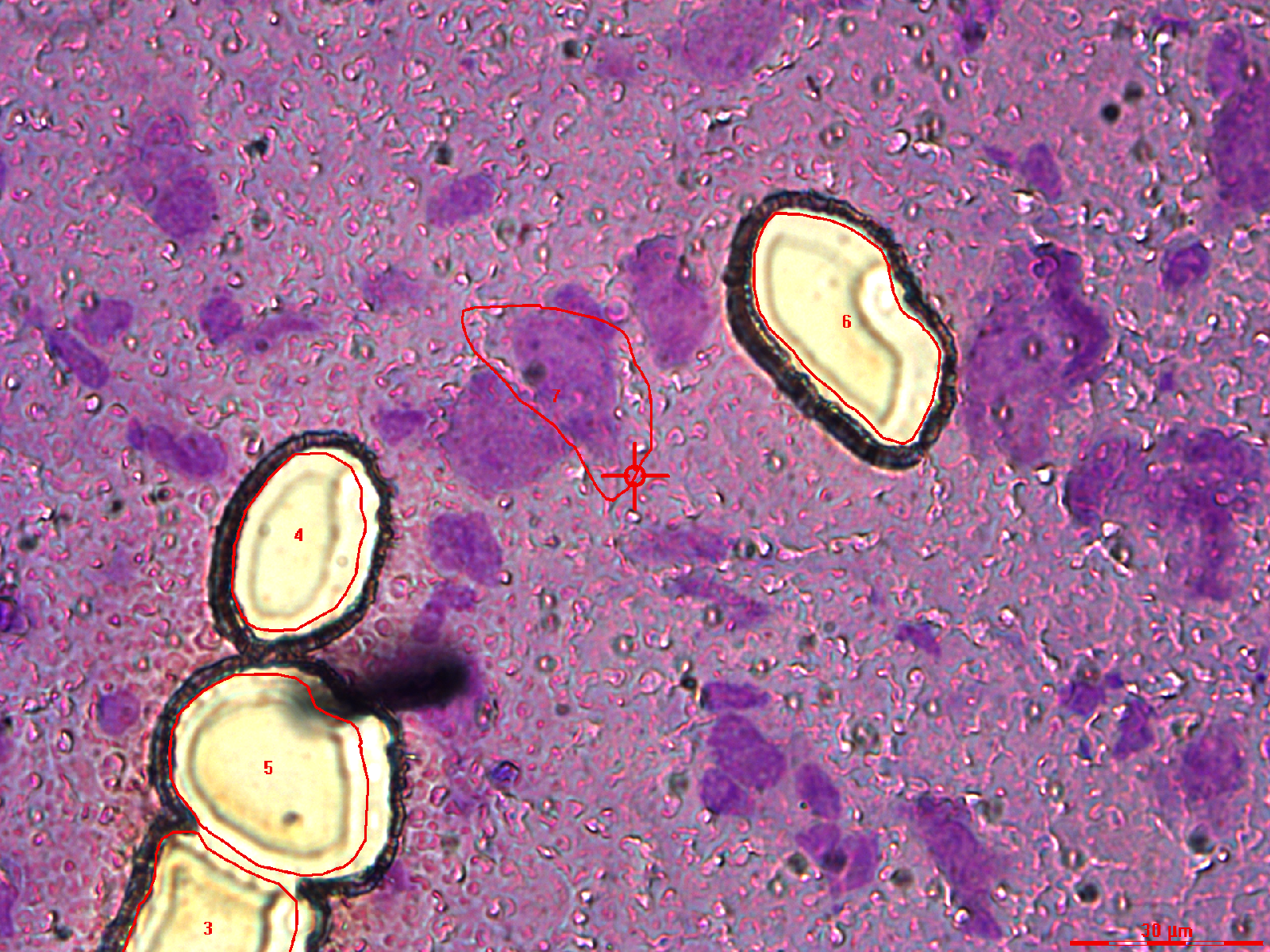

Supplement: Source data 1. [file elife-67464-data1.zip › Siller_et_al_2022/Figure_2-figure supplement 2_Alr_original.tif]

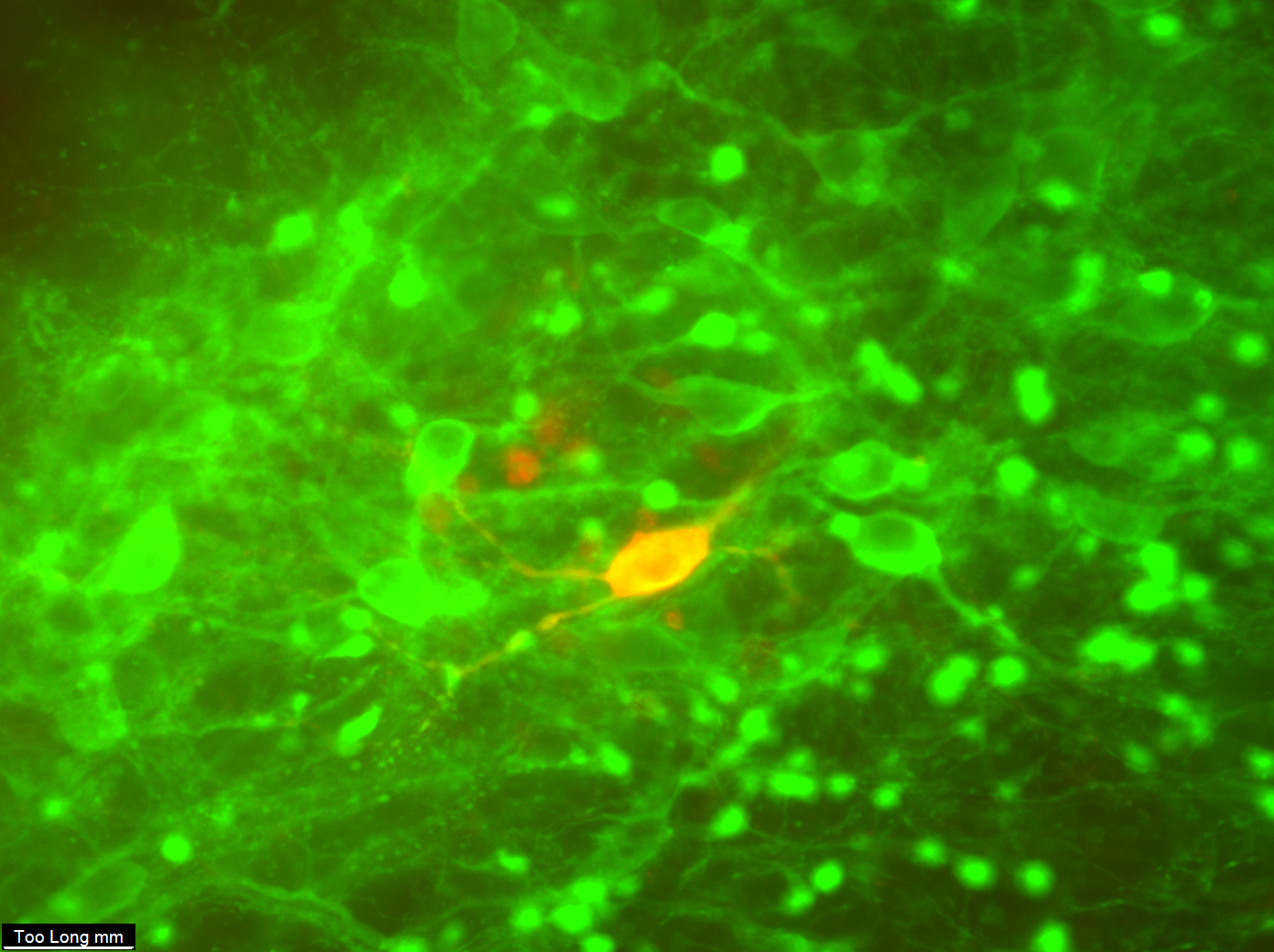

Supplement: Source data 1. [file elife-67464-data1.zip › Siller_et_al_2022/Figure_6_C_TH_NB_original overlay.png]

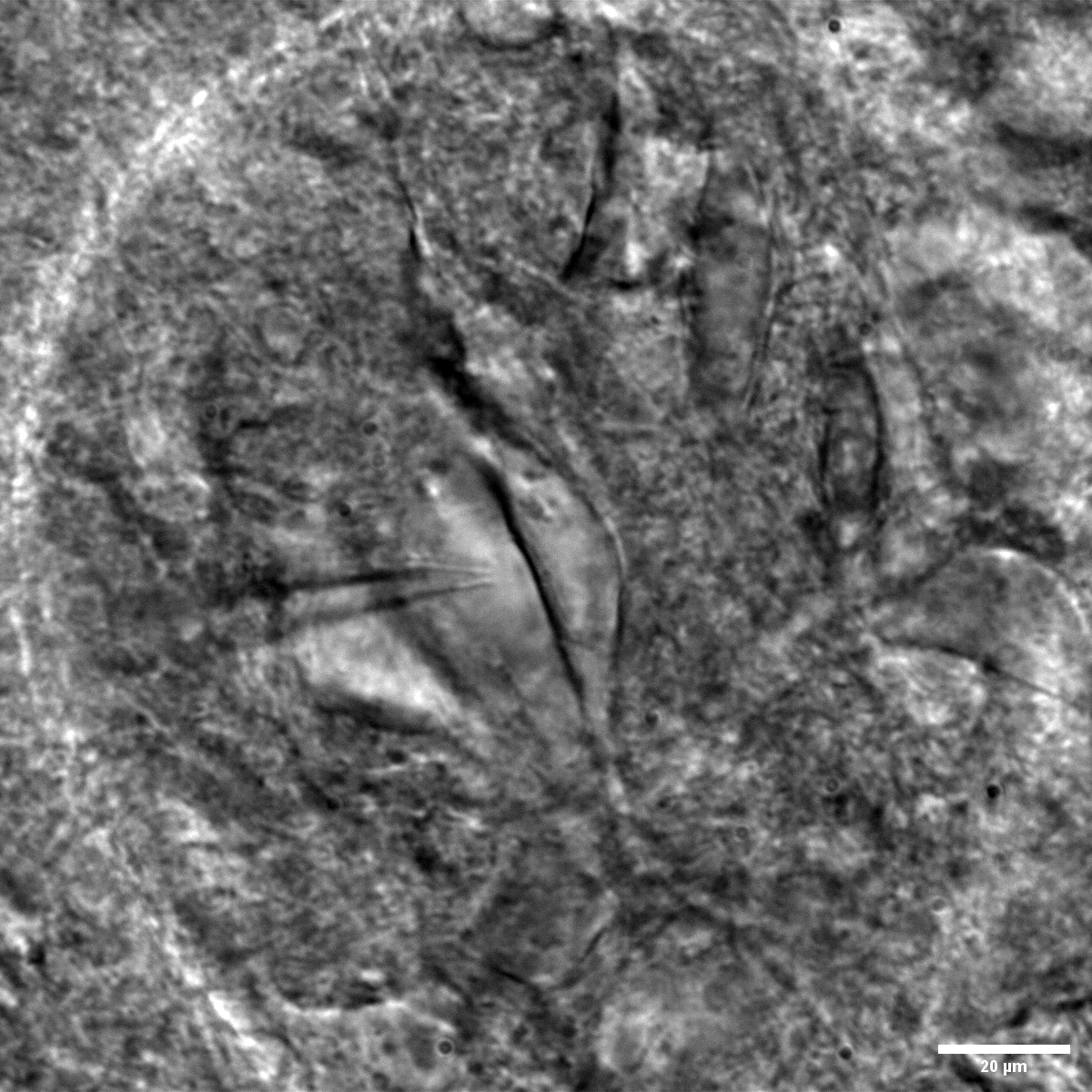

Supplement: Source data 1. [file elife-67464-data1.zip › Siller_et_al_2022/Figure_6_C_BF_original.png]

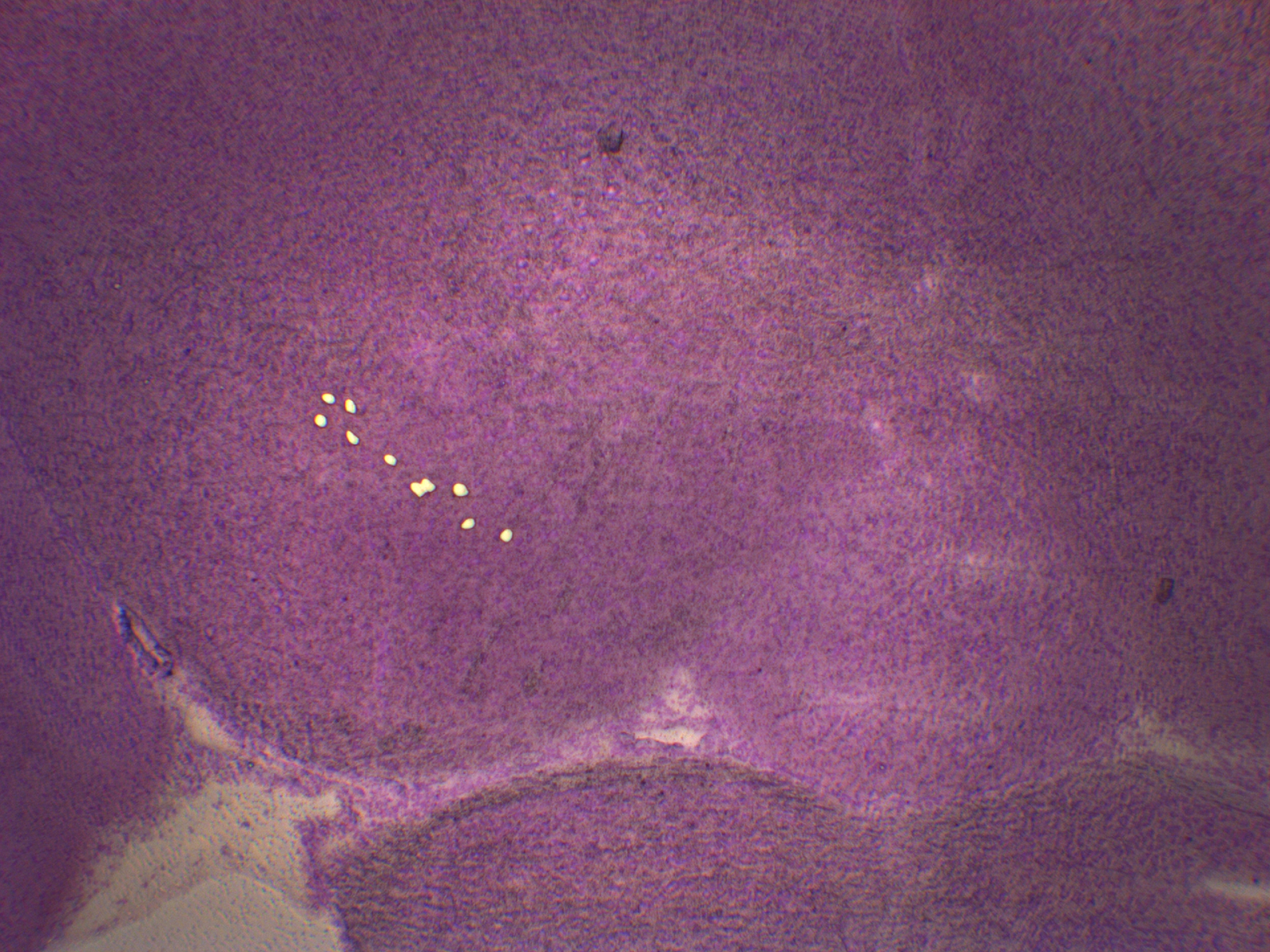

Supplement: Source data 1. [file elife-67464-data1.zip › Siller_et_al_2022/Figure_2-figure supplement 2_Aul_original.tif]

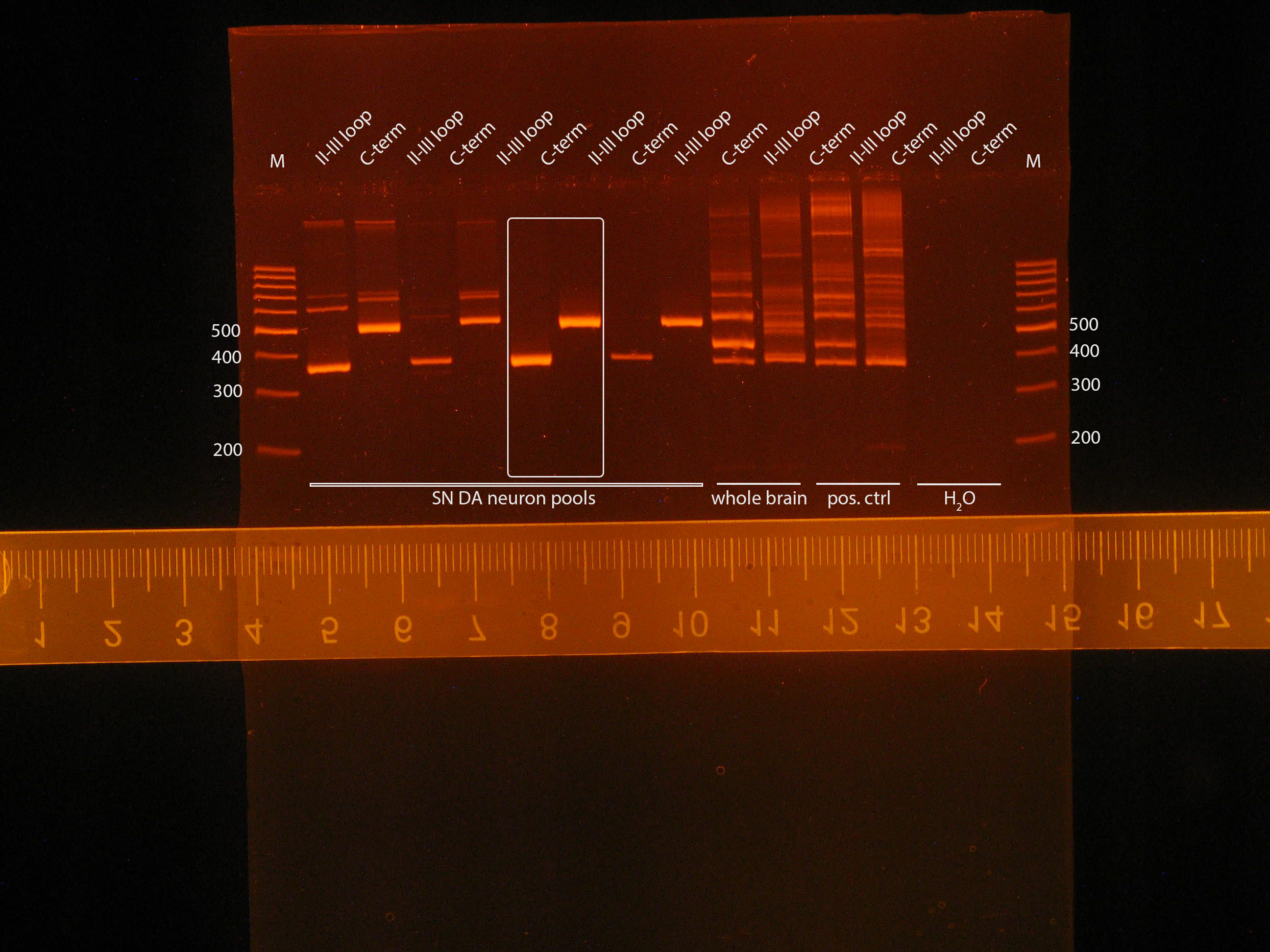

Supplement: Source data 1. [file elife-67464-data1.zip › Siller_et_al_2022/Figure_1-figure supplement 1_Bl_original_labelled.tif]

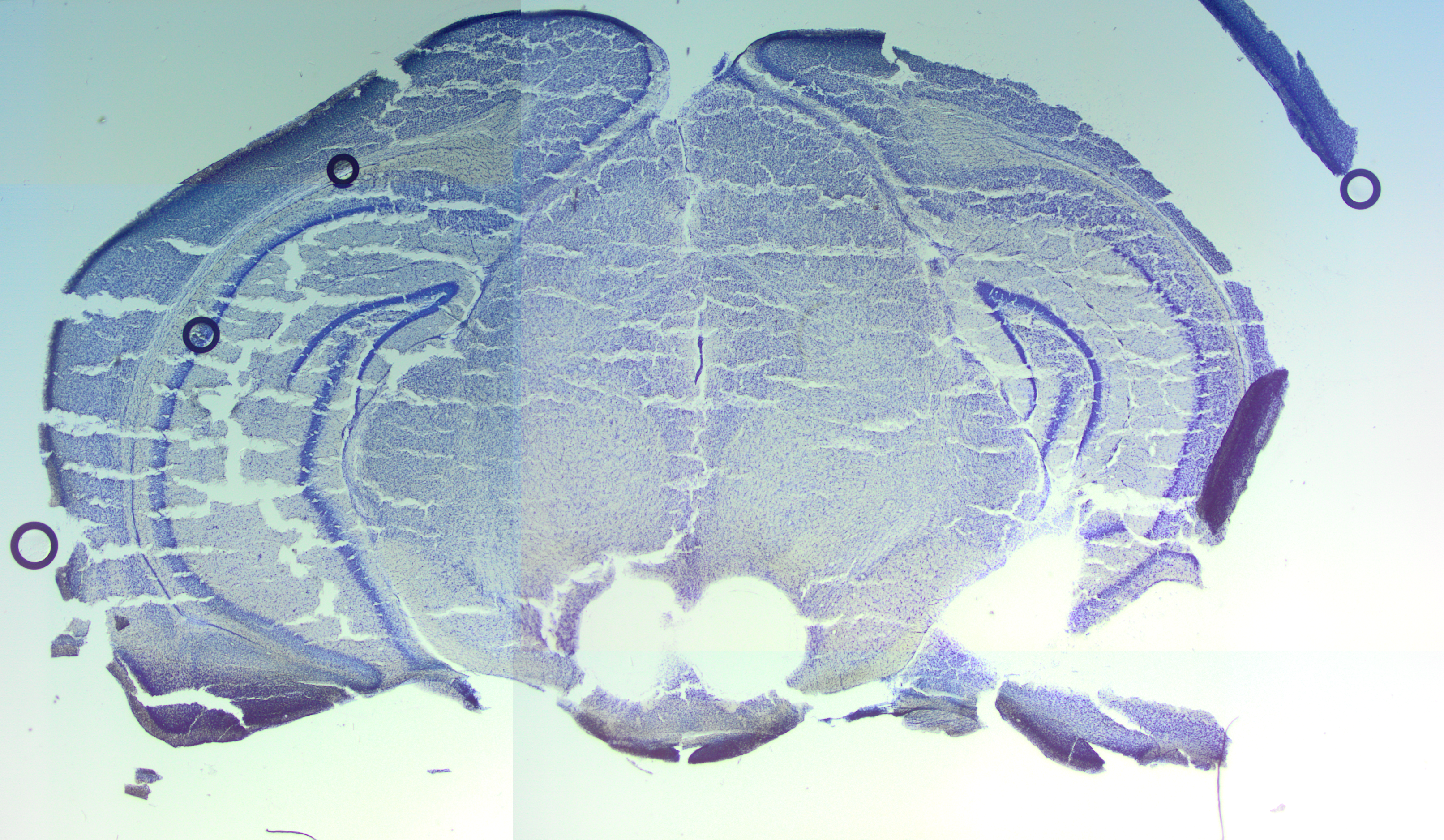

Supplement: Source data 1. [file elife-67464-data1.zip › Siller_et_al_2022/Figure_2_Cr_original.tif]
